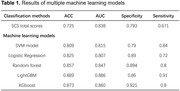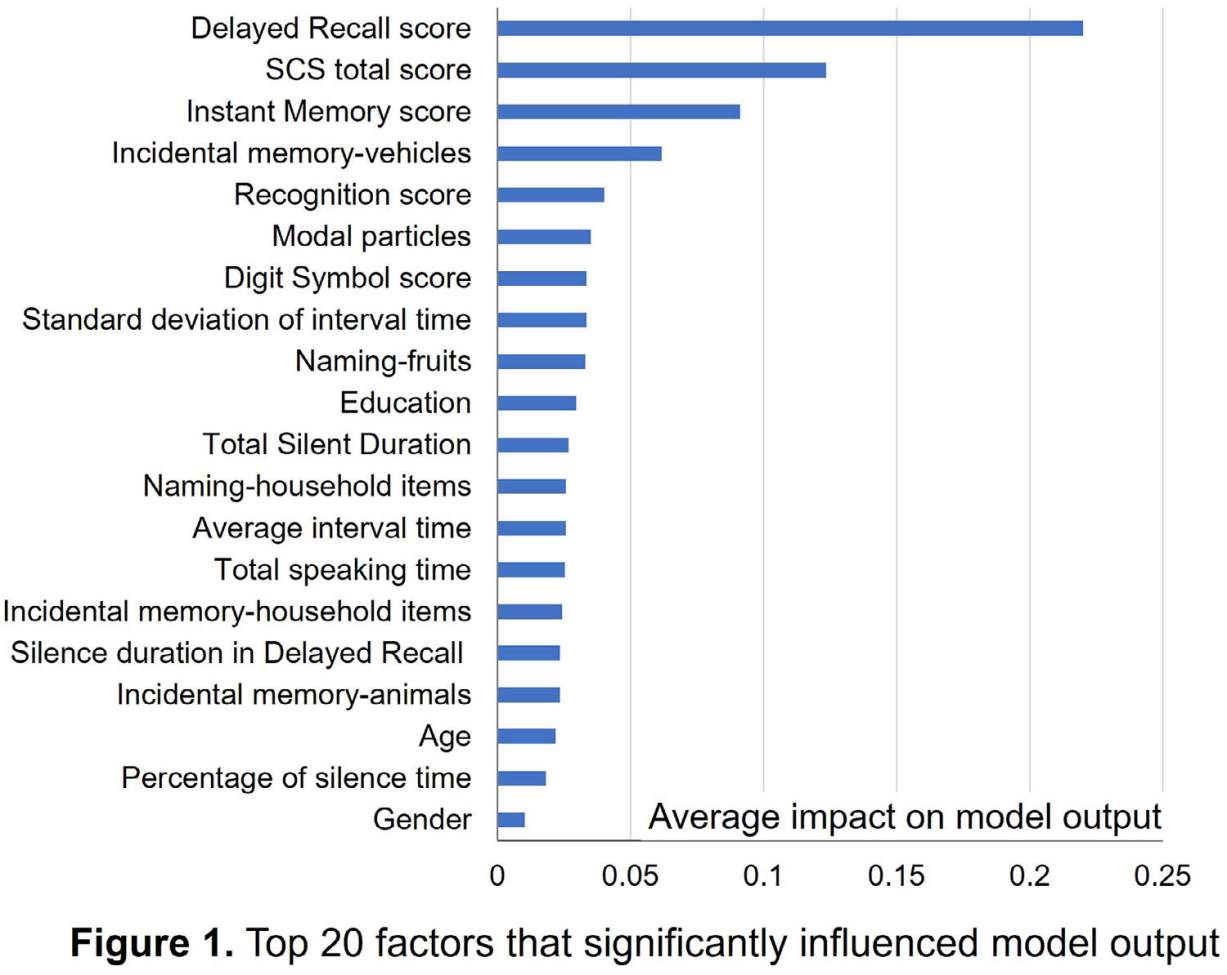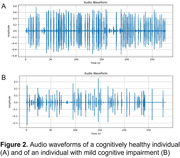# Machine Learning‐Driven Speech Biomarker Analysis: A Novel Approach for Detecting Cognitive Decline in Older Adults

**DOI:** 10.1002/alz.089668

**Published:** 2025-01-09

**Authors:** Yatian Li, Quan Chen, Jingnan Wu, Nan Chen, Zhixing Zhou, Huanhuan Xia, Lin Huang, Qihao Guo

**Affiliations:** ^1^ Shanghai Bestcovered Limited, Shanghai China; ^2^ School of Optical‐Electrical and Computer Engineering, University of Shanghai for Science and Technology, Shanghai China; ^3^ Shanghai Sixth People's Hospital Affiliated to Shanghai Jiao Tong University School of Medicine, Shanghai China

## Abstract

**Background:**

Speech impairment appears at early stages of Alzheimer’s disease. A mobile voice recognition‐based cognitive assessment tool, Shanghai Cognitive Screening (SCS), was developed for detecting mild cognitive impairment (MCI) and dementia in the community. The objective of this study is to investigate speech biomarkers associated with cognitive impairments based on SCS, and to evaluate the diagnostic accuracy of speech feature‐based machine learning (ML) models for detecting MCI.

**Method:**

A total of 301 older adults were recruited to perform SCS assessments, with 135 of them were diagnosed with cognitive impairment (MCI and early dementia) and the remainder were cognitively normal. Speech features were extracted from SCS voice recordings and integrated in a dataset along with SCS scores and demographic indicators. The dataset was randomly divided into training and validation sets in a 4:1 ratio and trained in various ML models with predictive speech features examined.

**Result:**

Among the models trained, the ML models employing SVM model, Logistic Regression, Random Forest, LightGBM, and XGBoost demonstrated higher accuracy than original SCS scores (Table 1). A ML classifier using Mel spectrograms and multimodal data fusion exhibited the highest accuracy of 0.953 (AUC = 0.967, sensitivity = 0.933, specificity = 0.933). Further analysis of the LightGBM model revealed that speech markers significantly influencing the model's output included modal particles, standard deviation of interval time, silence duration, mean interval time, total speaking time, silence time in delayed recall test, and percentage of silence time (Figure 1). These findings align with the audio waveform characteristics (i.e., smaller amplitudes, longer intervals, greater rhythm variations, and increased silence, Figure 2) in individuals with cognitive impairments. However, the top three factors were scores of delayed recall and instant memory, as well as SCS total score.

**Conclusion:**

The findings demonstrated speech biomarkers associated with cognitive impairments, facilitating more accurate detection of early cognitive impairment with ML techniques. Validation studies on larger sample sizes is necessary.